# Conversant or clueless? Chlamydia-related knowledge and practice of general practitioners in Western Australia

**DOI:** 10.1186/1471-2296-9-17

**Published:** 2008-02-29

**Authors:** Meredith J Temple-Smith, Donna Mak, Jan Watson, Lisa Bastian, Anthony Smith, Marian Pitts

**Affiliations:** 1 Primary Care Research Unit, University of Melbourne, Melbourne, Australia; 2 Department of Health Western Australia, Perth, Australia; 3 School of Medicine, University of Notre Dame, Fremantle, Australia; 4 Department of Human Services, Victoria, Australia; 5 Australian Research Centre in Sex, Health and Society, LaTrobe University, Melbourne, Australia

## Abstract

**Background:**

A survey of Western Australia's general practitioners' (GPs') knowledge and practices relating to genital chlamydia infection was conducted in mid-2005, prior to a multi-media campaign which encouraged 15–24 year olds to seek chlamydia testing through their general practitioner (GP). The survey aimed to raise GPs' awareness of chlamydia in preparation for the campaign and to establish a baseline measure of their chlamydia-related knowledge and practices.

**Methods:**

All 2038 GPs registered on the Australian Medical Publishing Company's database as practising in Western Australia were sent a survey which covered clinical features of chlamydia, investigations, treatment and public health issues; 576 (29%) responded.

**Results:**

Most GPs were aware of chlamydia being common in the 20–24 year old age group, but less than half were aware that it is common in 15–19 year olds. GPs missed many opportunities for chlamydia testing in patients likely to be at risk of STIs, largely because they thought the patient would be embarrassed. It is of concern that public health responsibilities in relation to chlamydia, ie notification and contact tracing, were not undertaken by all GPs.

**Conclusion:**

Australia is currently piloting chlamydia screening. For this to be successful, GPs will need to maintain current knowledge and clinical suspicion about chlamydia, and be comfortable in asking and receiving information about sexual behaviours. Only then will GPs have a significant impact on curbing Australia's ever-increasing rates of chlamydia.

## Background

In Western Australia (WA), as in other parts of Australia, the occurrence of genital chlamydia infection (from now on referred to as chlamydia) has been increasing, with notifications and age-standardised rates almost quadrupling from 1591 to 5863 (82.6 to 284.6 per 100,000), between 1997 and 2006 [1]. A recent study conducted in Victoria, Australia, showed strong correlation between chlamydia notification and testing rates in both men and women, suggesting that increased testing would identify further chlamydia infections [2].

Left untreated, chlamydia can cause pelvic inflammatory disease, ectopic pregnancy, infertility and chronic pelvic pain [3]. Early diagnosis can be achieved through screening and has been shown to be of benefit in reducing such complications [4,5]. Furthermore, access to nucleic acid testing, self-collected or non-invasive sampling, and single-dose treatment for chlamydia has been available since the late 1990s, removing previous barriers to early detection and treatment.

Chlamydia occurs in most age groups, but is most commonly notified in those under 25 years. A recent prevalence study in family planning clinics in New South Wales, Australia, found an overall prevalence of 5.6% in 16–25 year olds [6]; only slightly higher than the 5% prevalence found in over 500 18–24 year olds presenting to GPs in Queensland, Australia [7]. While it is likely that sexually transmissible infections (STIs) form a small part of most Australian GPs' caseloads, in WA most cases of chlamydia are diagnosed in general practice (Unpublished data, WA Notifiable Infectious Disease Database, Mak, personal communication, 2007.) GPs have reported varying levels of commitment to chlamydia screening, and a lack of willingness to screen opportunistically [8]. A likely contributing factor is the fact that many GPs fail to take a sufficiently detailed sexual history with which to assess the patient's sexual health risk; this has been found in many studies, both within Australia overseas, over the last decade [9-13]. One recent Australian study found GPs, particularly female GPs, were more likely to take a sexual history if they perceived a patient to be in a high-risk category, regardless of their actual risk behaviour [8]. A gender difference between GPs has been observed in relation to chlamydia testing more generally, with female GPs significantly more likely to offer testing than males both in Australia and overseas [8,13,14].

In WA in 2004, 63% of chlamydia notifications were for 15–24 year olds [1]. In June 2005, the Department of Health, Western Australia (DoH WA) launched a multi-media campaign, *Chlamydia: most people haven't got a clue*, encouraging young people aged 15–24 years to seek chlamydia testing from their GP [15].

To ensure that they were adequately prepared to both respond to increased requests for tests and to initiate discussion about chlamydia testing, GPs were surveyed about their chlamydia knowledge and practices, and professional development was offered to GPs who wished to improve their skills in this area.

## Methods

This study received approval from the LaTrobe University Human Ethics Committee.

Six weeks before the campaign a survey was sent to all GPs registered on the Australian Medical Publishing Company's database as practising in WA to:

• raise GP awareness of chlamydia

• to establish a baseline measure of GPs' chlamydia-related knowledge and practices and

• to encourage GPs to reflect on their practice in relation to STIs in general.

The 12 page, 32 item survey included items on the clinical features of chlamydia, chlamydia-related investigations, treatment and public health issues. GPs were encouraged to participate through three "Fax Alerts" sent to each GP by DoH WA. The first was sent just prior to the survey mail out, the second approximately one week after the survey would have been received, and the third one week before the closing date for return of surveys.

Of the 2038 surveys mailed out, 21 were returned not completed as the GP had resigned or semi-retired. A further 16 GPs had left the practice or changed address. In all 576 GPs responded, a response rate of 29% (576/2001). Twelve surveys returned after the closing date were excluded as answers may have been biased by the campaign. The final analysis included 564 surveys.

Gold Standard Answers to survey questions were mailed to all GPs who completed the survey and to all GPs as part of the *Chlamydia: most people haven't got a clue *promotional package for health professionals (which also included an order form for posters, pamphlets and guide to chlamydia testing). GPs who chose not to complete the survey thus still received educational material on chlamydia and sexual health issues.

A comparison between the demographics of study participants and a pool of over 2000 vocationally registered Australian GPs suggested the participants of this study to be slightly skewed to include more females, but to be representative of age [16].

## Results

### Who completed the survey?

Marginally more male (51%) than female (48%) GPs completed the survey (gender data was missing for 1%). The majority of respondents were in the 35–54 year age group (15% were less than 35 years, 31% were aged 34–44 years, 29% were aged 45–54 years, 15% were aged 55–64 years, and 11% were over 65 years). Almost 75% of GPs responding to the survey practiced in urban areas. Nearly two-thirds of respondents had been working in general practice for 10–29 years (21% had worked for 0–9 years, 34% for 10–19 years, 27% for 20–29 years, 12% for 30–39 years and the remaining 5% for over 40 years).

About 50% of respondents stated that young people aged 15–24 years comprised 10–25% of their patient caseload with a further 19% reporting that this age group comprised more than 25% of their patient caseload. Many GPs who completed the survey performed sexual health consultations such as offering contraceptive advice (56% daily; 89% at least weekly) Pap smears (51% daily, 81% at least weekly) and safe sex advice (30% daily; 72% at least weekly). GP respondents diagnosed STIs regularly (21% at least weekly; 66% at least monthly) and recommended STI tests to asymptomatic 'at risk' patients (14% daily, 45% at least weekly and 70% at least monthly) on a regular basis.

Respondents reported diagnosing 266 cases of chlamydia in the four weeks before receiving the survey. The almost 75% of the total number of respondents who practiced in urban areas, diagnosed 72% of these cases.

### Sexual risk assessment

When seeing patients whom they believed to be at risk of STIs, 81% of GPs reported that they commonly or very commonly asked about safe sex, 66% about having more than one sex partner and 65% about injecting drug use. However, fewer GPs asked about the important risk factors of overseas travel (54%) or sex with sex workers (30%).

GPs were asked whether they would be likely to take or update a sexual history in five different clinical situations. Nearly all (96%) GPs would take a sexual history from a man presenting as the sexual contact of an infected partner, and around half would do so for a female patient requesting a Pap smear. However, only 39% would do so for a 24 year old woman routinely presenting for the contraceptive pill, and around a third would do so for a male patient requesting overseas travel immunisation advice (34%) or a young male with a new sexual partner (29%). GPs were also asked how embarrassed a patient would be if they took a sexual history in these situations. More than two-thirds thought the young male sexual contact of an infected partner (82%), and a young woman seeking a Pap smear would not be embarrassed (79%). However, over half of respondents believed a woman seeking a prescription for the contraceptive pill (72%), a young man with a new girlfriend (58%) and a man seeking overseas travel immunisation advice (57%), would be embarrassed or very embarrassed by the GP taking a sexual history.

There was a clear gender bias in terms of sexual risk assessment with significantly fewer female than male GPs believing that the female patients would be embarrassed if they were to take or update a sexual history. Similarly significantly fewer male than female GPs believed that the male patients would be embarrassed if they were to take or update a sexual history (Table 1). Despite this, female GPs were generally more likely report that they would take or update a sexual history (Table 2).

**Table 1:** Percentage of GPs who believed that these patients would be embarrassed or very embarrassed if they were to take a sexual history, by gender of GP

	**Female **n = 268	**Male **n = 284	χ^2 ^p value
24 year old woman requires prescription for contraceptive pill	14.4	39.4	<0.001
24 year old woman for Pap smear test	11.1	30.1	<0.001
45 year old man requests travel immunisation advice	53.1	31.2	<0.001
32 year old man whose girlfriend has a vaginal infection	23.0	14.3	0.01
20 year old man for asthma medication prescription who also has new girlfriend	49.8	33.3	<0.001

**Table 2:** Percentage of GPs who are likely or very likely to take or update a sexual history, by gender of GP

	**Female **n = 268	**Male **n = 284	χ^2 ^p value
24 year old woman requires prescription for contraceptive pill	51.8	27.1	<0.001
24 year old woman for Pap smear test	71.6	39.0	<0.001
45 year old man requests travel immunisation advice	32.3	34.8	NS*
32 year old man whose girlfriend has a vaginal infection	97.3	94.0	0.05
20 year old man for asthma medication prescription who also has new girlfriend	32.0	25.7	NS*

* Not significant at 0.05 level

GPs were asked how they would rate the likelihood of their recommending a chlamydia test in five different clinical situations (Table 3). Most GPs (females, 96%; males 93%) reported that they would do so for the young male presenting as the sexual contact of someone with a vaginal infection. Female GPs were more likely than males to recommend chlamydia testing for the female patients requesting contraception (37% vs. 14%, χ^2 ^p < 0.001) and a Pap smear (70% vs. 30%, χ^2^, p < 0.001), (Table 3). Differences in responses depending on age and rural/urban status were evident but minor in comparison to gender differences.

**Table 3:** Percentage of GPs who are likely or very likely to recommend testing for chlamydia by gender and age of GP

	**Female**	**Male**	**25–34 years**	**35–44 years**	**45–54 years**	**55–64 years**	**65+ years**
	n = 268	n = 284	n = 61	n = 164	n = 187	n = 101	n = 44

24 year old woman requires prescription for contraceptive pill	36.9	14.4	29.5	29.8	24.0	24.7	9.0
24 year old woman for Pap smear test	70.1	30.3	67.2	58.5	46.5	38.6	31.8
45 year old man requests travel immunisation advice	8.5	10.5	11.4	6.7	9.0	10.8	13.6
32 year old man whose girlfriend has a vaginal infection	95.8	93.3	98.3	95.7	93.5	94.0	88.6
20 year old man for asthma medication prescription who also has new girlfriend	23.8	20.7	24.5	21.9	22.9	19.8	22.7

For patients presenting with STI symptoms, most GPs (87%) reported that they would commonly ask about a previous STI history, 65% about injecting drug use, 67% about recent overseas travel and 55% about specific sexual practices.

### Knowledge of age-groups in which chlamydia mostly is notified

Both nationally and in WA, chlamydia is most commonly seen in the 15–19 year and the 20–24 year age-groups. When asked which were the main age groups in which chlamydia is most commonly seen, 76% of respondents selected the older group, while less than half (45%) selected the younger group. Almost 30% of respondents selected the 25–29 year old age group, 4% selected the 30–34 year old age group, and 2% selected the 35–39 year old age group. Less than 1% believed chlamydia occurred most commonly at no particular age.

### Public health responsibilities

Almost all GPs (99%) knew that chlamydia is a notifiable infection in WA; however, only 85% stated that they would always complete a notification form.

Contact tracing practices were variable, with less than 25% of respondents considering this to be always or mostly their responsibility (Table 4). Slightly over half (51%) of the participants sometimes considered contact tracing to be their responsibility, whilst 21% believed this never to be the case. In fact, in a patient in whom respondents had diagnosed a laboratory confirmed STI, only 60% would commonly ask details of sex partner for contact tracing purposes.

**Table 4:** Percentage of total sample (n = 576) who consider that contact tracing is their responsibility

Always	7.4
Mostly	17.2
Sometimes	51.6
Never	21.3
Missing	2.5

## Discussion and Conclusion

Information on the sexual health practices of the WA GP workforce are not available, so it is impossible to determine how representative the survey respondents are of the GPs in WA. Over half of the respondents were performing Pap tests and providing contraceptive advice daily. This suggests that GPs returning the survey had some interest in reproductive health issues, and thus could reasonably be expected to have a better than average knowledge of sexual health.

It is likely that low STI caseloads were one reason for non-response to the survey, as was found in a recent chlamydia-related study of GPs in New South Wales with a 45% response rate [17]. These findings also suggest that our survey was returned by GPs with at least some interest in sexual health.

Whilst the modest return rate of 29% is clearly a limitation of the study, a recent postal survey of GPs in south-eastern Australia, with a nearly 60% response rate, reported very similar findings [18]. The response rate in our study also holds a warning that non-responders may have poorer chlamydia-related knowledge and practices than respondents. This might not be of great concern if all respondents had excellent knowledge and exemplary practices. However, amongst responders to the survey, most of whom are actively engaged in reproductive and sexual health services, there are still many findings of concern.

Foremost among these were GPs' practices in relation to public health more generally. Whilst almost all GPs knew that chlamydia was notifiable, some GPs stated that they do not usually notify DoH WA of such infections. Less than 25% of GPs saw contact tracing as their responsibility, with a third not even usually asking a patient for details about their sex partners for contact tracing purposes. While general practice is not well structured for contact tracing, this responsibility may benefit from simple innovative interventions, some of which are being piloted as part of the pilot testing program for chlamydia. These include contact tracing practice for practice nurses, and a message on the laboratory result of positive chlamydia tests providing the notifying doctor with a website containing chlamydia treatment guidelines, client brochure, and a printable letter for index cases to pass on to exposed sexual partners [19].

Results of the study suggested that GPs may be missing opportunities to assess the likelihood of chlamydia in many of those most at risk. They thus need to maintain a high level of clinical suspicion to consider a chlamydia diagnosis. Even when GPs do initiate STI testing, many do not ask all the questions necessary to ensure that appropriate STI tests are performed, eg. specific sexual practices and injecting drug use which may indicate a greater likelihood of BBVs.

Although GPs generally asked the most important questions of patients with an obvious STI risk, other opportunities where sexual risk assessment could be easily justified to the patient, such as during a Pap smear test and in a consultation about contraception, were not as readily taken up, particularly by male GPs. Yet Khan et al found over half of the GPs in their study felt chlamydia testing should be offered during a consultation at which a Pap smear was taken [8].

Targeted screening for chlamydia requires considerable communication skills [14]. A randomised controlled trial comparing computer-assisted with face-to-face sexual history taking in a sexual health centre showed that women reported significantly higher numbers of male partners in the preceding 12 months when completing a computer assisted self interview, suggesting that patients require a non-judgemental environment to answer honestly questions about such sensitive issues [20]. A recent study showing a positive association between chlamydia prevalence in young women and the numbers of male sexual partners in the preceding year underscores the importance of assessing the true numbers of sexual partners [21].

Given also that sexual history taking in general practice is not commonly performed and frequently inadequate [9-13], the challenge remains to ensure that neither asking nor answering questions about sexual behaviour causes embarrassment or shame for GPs or their patients.

With widespread availability of nucleic acid testing and safe, effective single-dose treatment, chlamydia is an epidemic which should be addressed not just by opportunistic case-finding but by a comprehensive population-based control program. The effectiveness of screening for chlamydia in asymptomatic young women has been shown in studies of screening programs in Sweden and the USA [22-25]. These countries have different health care systems to Australia where most chlamydia is diagnosed in general practice. However a recent analysis of annual opportunistic screening in women under 25 suggested that chlamydia screening would be cost-effective in Australia [26]. Such screening, based on age and ever-had-sex that was appropriately advertised and funded would overcome the discomfort and shame felt by many young people about being asked about their sexual practices, and by many GPs whose job it is to ask these questions.

If it is indeed true that the respondents to this survey represent the GPs who have some interest in sexual health and/or a higher caseload of young people, then there is much work ahead if all GPs are to be adequately trained in best practice for chlamydia screening. GPs can make a significant impact on chlamydia control through both individual risk assessment and screening. However, it is critical that GPs have the knowledge and skills to achieve this. If three things can be achieved – current knowledge, clinical suspicion, and comfort in asking about sexual behaviours – we will be well on our way to gaining control over the current chlamydia epidemic.

## Competing interests

The author(s) declare that they have no competing interests.

## Authors' contributions

JW analysed data; MT-S and JW wrote first draft; all other authors commented on various drafts, and all authors read and approved the final version.

## Pre-publication history

The pre-publication history for this paper can be accessed here:


